# Microplastics through the Lens of Colloid Science

**DOI:** 10.1021/acsenvironau.1c00016

**Published:** 2021-09-10

**Authors:** Ahmed Al Harraq, Bhuvnesh Bharti

**Affiliations:** Cain Department of Chemical Engineering, Louisiana State University, Baton Rouge, Louisiana 70803, United States

**Keywords:** microplastics, nanoplastics, colloids, pollutant adsorption, dynamic wettability, interparticle interactions

## Abstract

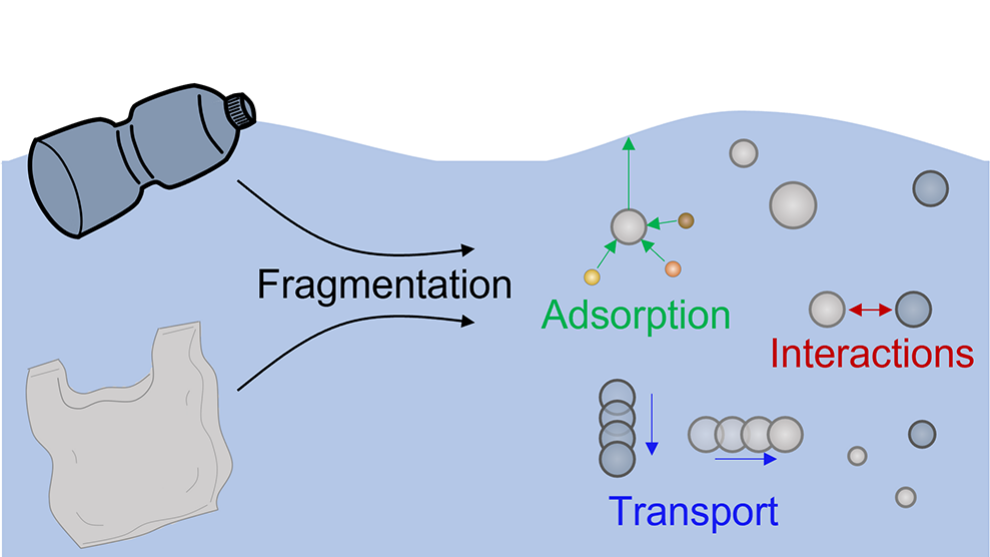

Microplastics are sub-millimeter-sized fragments of plastics and a relatively new class of pollutant increasingly found in the environment. Due to their size and surface area to volume ratio, the physicochemical characteristics of microplastics can diverge from those of their macroscopic counterparts. This is partly why it is challenging to understand their origin, analyze their behavior, and predict their fates in the environment compared to large pollutants. We believe that adopting a view of microplastics as a colloid provides a holistic framework that connects their physical properties and surface chemistries with observations of their dynamics in the environment. In particular, we discuss the role of fundamental principles of colloid science in interpreting phenomena of wetting, adsorption, aggregation, and transport of microplastics. Colloid and interface science can provide the tools to couple or decouple the physicochemical behaviors of microplastics, which may aid in understanding the environmental challenge both from a fundamental perspective and with respect to practical remediation methods.

## Introduction

The environmental threat of plastic pollution is predicted to worsen over the next decade, with growing waste disposal in aquatic ecosystems.^1^ The issue of plastic pollution is further amplified by reports of widespread presence of tiny fragments of plastics not only in beaches, marine, and freshwater bodies^2^ but also in remote areas such as deserts,^3^ mountain ranges,^4^ and polar regions.^5^ These so-called microplastics are particles typically sized between a few nanometers (also referred to as nanoplastics) and several millimeters, which pose unique risks to the environment and human health. Microplastics are hardly visible to the naked eye, and their classification and quantification in the environment are more complex than their macroscopic counterparts.^6^ The small size of microplastics renders this synthetic matter ingestible by marine biota, thus potentially intoxicating aquatic creatures and climbing up the food chain. Microplastics may act as a vehicle for organic and heavy metal pollutants to enter the human food web, due to the tendency of such chemicals to adsorb on plastics.^7,8^

**Figure 1:**
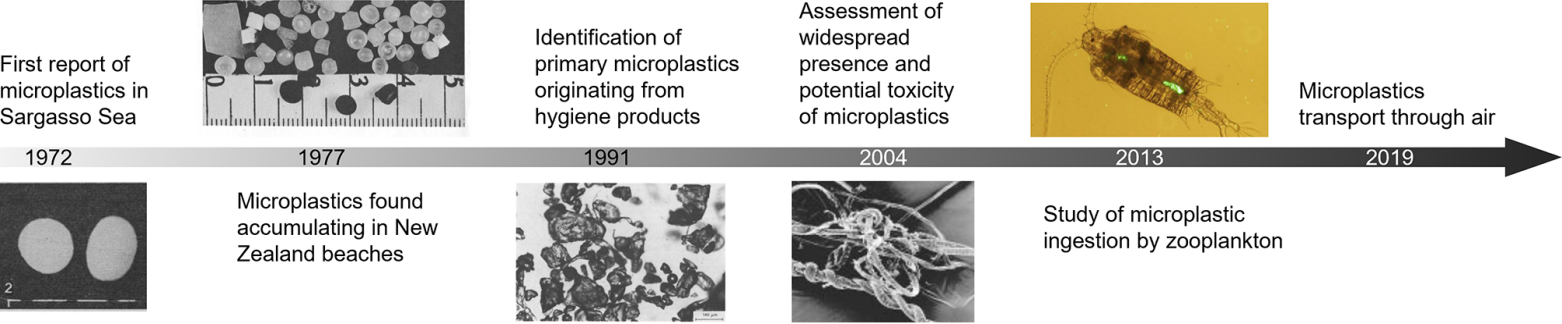
Summary of some major steps in the history of research in microplastics. Images appearing from left to right are reproduced with permission from refs (9) (copyright 1972 AAAS), (12) (copyright 1977 Elsevier), (13) (copyright 1991 Elsevier), (14) (copyright 2004 AAAS), and (16) (copyright 2013 American Chemical Society).

Initial reporting of the presence of microplastics began in the 1970s when plastic beads of various chemical nature were found on the surface of North Atlantic waters^9−11^ and New Zealand beaches.^12^ A distinction emerged between primary and secondary microplastics: the former are introduced to the environment as sub-centimeter-sized particles used in cosmetic and hygiene products,^13^ whereas the latter are formed in the environment via weathering and degradation of large plastic waste. The accumulation of reports has been reviewed extensively,^2,14,15^ particularly since the 2000s, with increasing evidence of the threat to the environment posed by microplastics. While the crisis is inextricably linked to the wider issue of plastic waste, the microscopic nature of these pollutants differentiates their physicochemical properties from their macroscopic counterparts. In the past 10 years, a new research direction has emerged with focus on analyzing the specific features of microplastics, such as the interaction with aquatic life^16^ and modes of transport.^4,17^ Some key historical elements of microplastics research are summarized in Figure 1.

**Figure 2:**
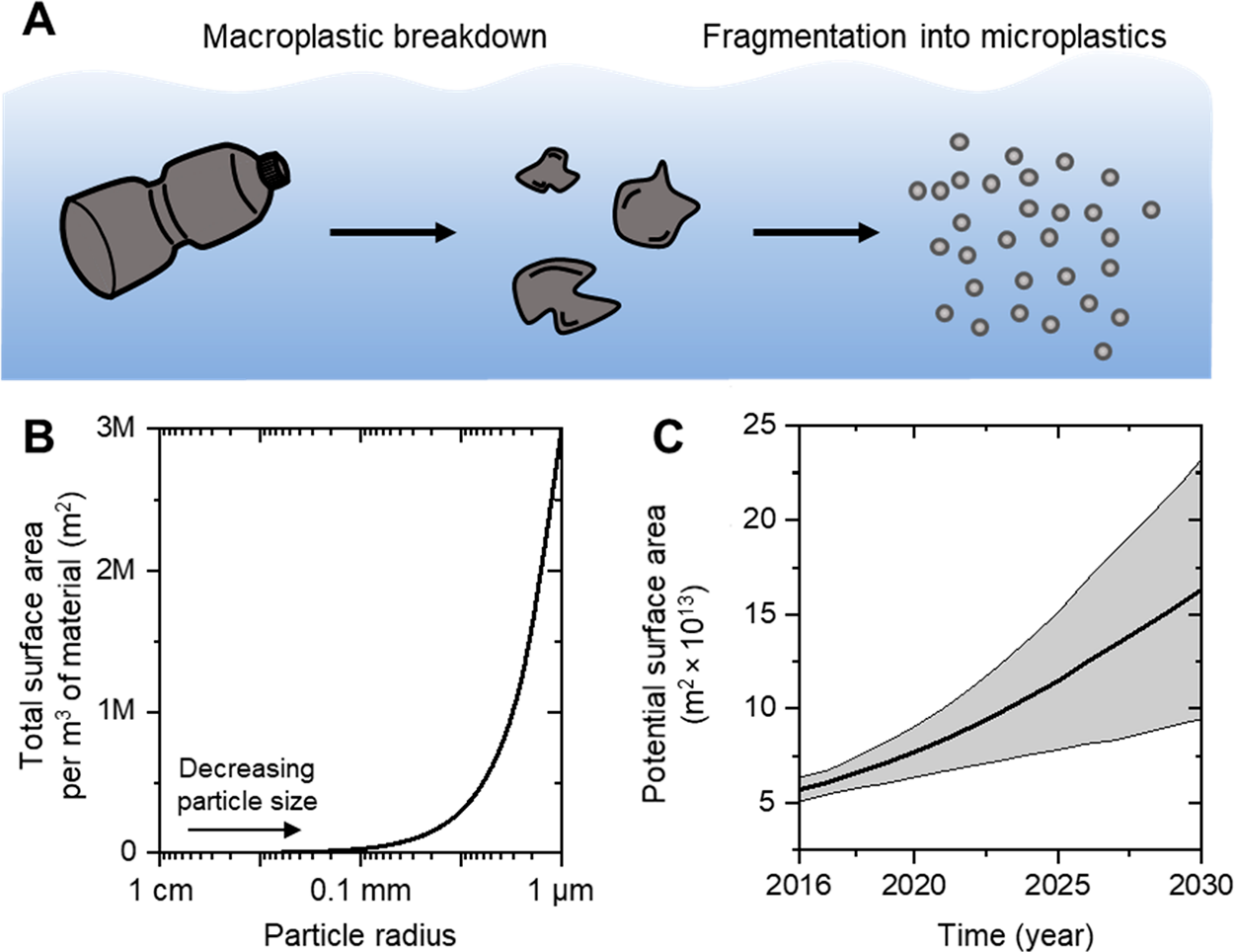
(A) Schematic of microplastic breakdown in the environment. (B) Dependence of surface to bulk ratio on particle size. As particles approach the colloidal length scale, the surface area per volume of material increases dramatically. (C) Plot of potential surface area of microplastics introduced in the environment annually. The values are obtained assuming breakdown of large plastic waste into 1 μm sized microplastic fragments. The original data are from Borrelle et al.^1^

As plastic waste is introduced in the environment, weathering induced by physical abrasion and hydrolysis coupled with photo-, thermal, and biodegradation leads to the breakdown of macroplastics and its fragmentation into microplastics (see Figure 2A). Plastic litter on sandy beaches, in particular, is subjected to both high heat and high UV loads over time.^18^ This is shown to cause microcracking of the surface, rendering the material brittle and susceptible to fracture.^19^ Sand abrasion and other environmental stresses eventually result in the formation of microplastics.^20^

Plastic fragmentation drives an exponential increase in the interfacial area and changes in the surface chemistry (Figure 2B), which must be accounted in predicting the environmental fate of microplastics. Recent estimates of global plastic emissions in aquatic environments show an unsustainable increase over the next 10 years, especially in the “business as usual” scenario.^1^ Though microplastic formation “lags” behind the introduction of plastics in the environment, we can estimate that societal waste adds up to ∼10^13^ m^2^ of potential debris surface annually (Figure 2C). These estimates are based on the recent study by Borrelle et al.^1^ and assuming that all plastic eventually breaks down into spherical microplastics of diameter 1 μm with a mass density of ∼1144 kg m^–3^.

Most differences between macro- and microplastics stem from the following basic principle: the interfaces of materials have different properties from their bulk, and the smaller a material gets, the higher its surface area to volume ratio becomes. This is the realm of colloid science which deals with particles immersed in a medium, in the size range of microplastics, that is, from nanoscopic to several microns in diameter. At these length scales, particles are small enough to be susceptible to thermal fluctuations, thus undergoing Brownian motion, but are indifferent to quantum effects. Micro- and nanoscopic colloids interact across their surrounding medium, often leading to the clumping or aggregation of particles. One of the properties that acquires a fundamental role at small scales is surface tension, which is often responsible for the floating of tiny plastic debris. Similarly, high interfacial areas increase the extent of adsorption occurring on plastics, amplifying the role of surface over bulk chemistry. Furthermore, colloidal particles have “electrical double layers” (EDL) originating from the separation of charges on the surface and Coulombic attraction of oppositely charged ions in an aqueous solution. As a result, charge effects arising from variations in salinity and pH play a role exclusive to microplastics that is not relevant to large plastics. Analogously, interfaces are subject to the photochemical effects which points to a potential larger impact of sunlight on microplastics than on macroplastics. Lastly, the transport of small colloids is varied in modality, with current flow, sedimentation, and also atmospheric transport affected by the size and surface properties of the microplastics. These are a few examples that point to the divergence in physical chemistry between macro- and microplastics with fundamental consequences over environmental fate.

The urgency of the problem requires the contribution of laboratory experiments that can be combined with field studies to understand the fate and impact of microplastics in the environment. This involves the application of a number of analytical tools via protocols that have been developed in recent years. For example, Mintenig et al. have proposed a framework of techniques that include sampling via filtration methods, sizing via asymmetrical flow field-flow fractionation, and characterization via micro-FTIR and mass spectroscopy.^21^ We showcase some ways in which colloid and interface science are of direct relevance in understanding, analyzing, and potentially predicting the physical behavior of microplastics in the environment. Of the many intersectional questions, we selected to discuss the role of wettability, the potential for adsorption, the effect of particle interactions, and some relevant principles of colloidal transport. The conclusion will outline how we expect the contribution of colloid science to impact both our fundamental understanding of microplastics and our ability to make predictions on their environmental fate.

## Wettability and Adsorption

**Figure 3:**
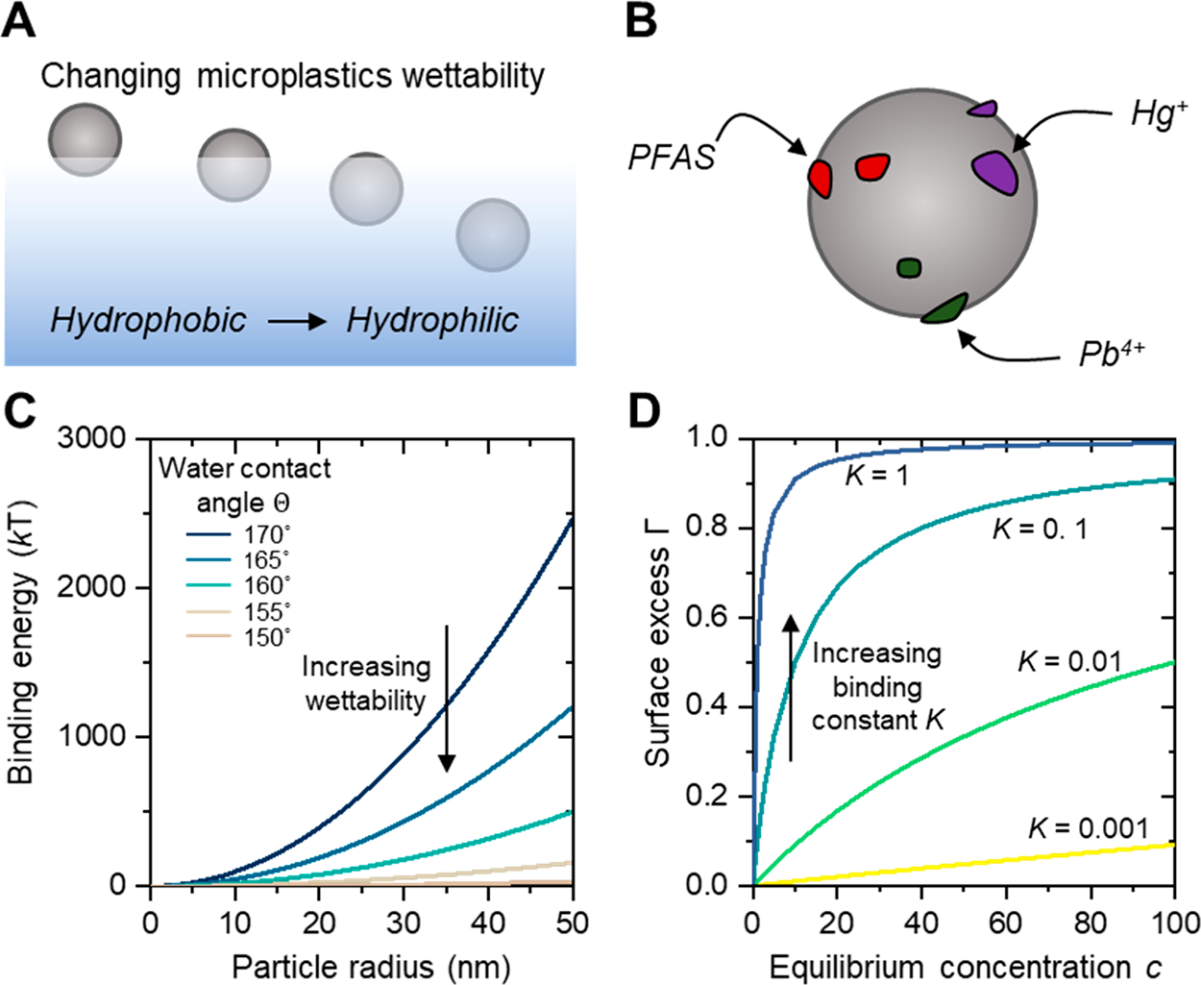
(A) Hydrophobic-to-hydrophilic transition of microplastics surface can occur in water. Changes in wettability can lead to the dispersion of particles from the water surface to the bulk. (B) Schematic of adsorption of pollutant molecule, such as perfluoroalkyls (PFAS), mercury (Hg^+^), and lead (Pb^+^) ions, on the surface of a microplastic particle. (C) Binding energy of a particle decreases nonlinearly with decreasing radius and wettability. As microplastics decrease in size and become more hydrophilic, they are more likely to unbind from interfaces. (D) Pollutant molecules adsorb more drastically to a surface with high binding constant.

Wettability indicates the affinity for a liquid to form a stable interface with a solid, and it originates from a balance of adhesive and cohesive molecular interactions (Figure 3A). The vast majority of plastic waste is made of hydrophobic polymers such as polyethylene (PE), polypropylene (PP), and polyvinyl chloride (PVC), which means that they have a natural tendency to separate from water molecules and thus not water-wet. The “dislike” of water combined with the low mass density of microplastics could have two major implications on their behavior: (1) binding of microplastics to liquid–vapor or solid–liquid interfaces and (2) adsorption of foreign molecules onto the surface of microplastics (Figure 3B). Both of these effects are the result of minimization of interfacial free energy, where the interfacial adsorption of/onto microplastics decreases its contact area with the solvent.

The wettability of macro- and microplastics is dependent on the nature and number density of the chemical functional groups on the surface. It becomes increasingly important as the size of microplastics decreases, and it is one the major factors governing the properties of the microplastics in the environment. The binding energy (Δ*E*) of a spherical microplastic particle to an air–water interface is given as Δ*E* = −π*R*^2^γ(1 – |cos Θ|)^2^, where γ is the air–water surface tension, Θ is the contact angle of water on the surface of microplastics, and *R* is the radius of the microplastic particle.^22,23^ The nonlinear decrease of Δ*E* with decreasing particle radius and water contact angle at constant γ is shown in Figure 3C. The calculations indicate that decreasing size and increasing wettability of particles results in a decrease in the interfacial binding energy of the microplastic particles, pointing to a potential increase in dispersibility of the microplastics. Both the size and wettability of microplastics can be significantly influenced by natural weathering of discarded plastic waste (discussed below). Thus, relating microplastic aging with their dispersibility is necessary, but so far it has been overlooked.

Wettability of microplastics can be significantly influenced and altered by the environmental parameters. The wettability of microplastics may change due to chemical transformations in the plastics and/or molecular adsorption on the surface of nanoparticles.^24^ The dynamic nature of wettability changes poses a challenge to our ability to predict the fate of microplastics in the environment. One potential mode of altering the surface chemistry and wettability of macroscopic plastics is photo-oxidation. It is the major route for polymer degradation in the environment, as UV light can produce free radicals that catalyze the decomposition of several surface groups responsible for the hydrophobicity of the material.^25−27^ It is tempting to argue that known increase in hydrophilicity/water wettability of macroscopic plastic could imply an increase in dispersibility of microplastic, but no such focused study currently exists.

The binding of hydrophobic particles onto external interfaces may enable their transport to unexpected areas and/or sink to the bulk of oceans or lakes.^28^ Interestingly, the nonwettability of commodity polymers is exploited to collect microplastics from water for analysis, while separating it from hydrophilic sand.^29^ Briefly, a separation column filled with water containing microplastics and sand is bubbled with air introduced from the bottom. Due to their hydrophobicity, microplastics attach to the air–water interface and travel upward through the column, separating from the rest of the sediment. A similar concept has been proposed as a potential method to remove microplastics from the environment, using a highly hydrophobic substrate on which plastic debris would attach while water and other sediment particles do not.^30^

The surface chemistry of microplastics governs the adsorption of foreign molecules on its surface. The binding of matter onto interfaces is represented in the form of adsorption isotherms. Here, the amount of adsorbate molecules bound onto an adsorbent surface is determined as a function of equilibrium bulk concentration of adsorbate at constant temperature. One of the simplest and most widely used theoretical models representing molecular adsorption onto interfaces was proposed by Irving Langmuir.^31^ The major assumptions of the Langmuir adsorption model are the following: all adsorbing sites on the adsorbent are equivalent; the adsorbate forms a monolayer on the adsorbent; all adsorbate–adsorbate interactions are negligible. Under these assumptions, the amount adsorbed (Γ) is represented as Γ = Γ_m_*Kc*/(1 + *Kc*), where *c* is the equilibrium concentration of the adsorbate in bulk medium, Γ_m_ is the maximum surface concentration of the adsorbate, and *K* is the adsorption constant.^32^ For adsorption processes occurring in aqueous media, the adsorption constant *K* is related to the binding free energy as Δ*G*_ads_ = −*N*_A_*kT* ln(55.5*K*), where *N*_A_ is Avogadro’s constant, *k* is the Boltzmann constant, and *T* is the temperature.^33^ The adsorption free energy is dependent on the surface chemistry of the microplastics and changes depending on its weathering state and chemical composition. The change in the adsorption behavior according to the Langmuir model with increasing binding constant *K* is shown in Figure 3D. The larger binding energy shows a steep increase of surface concentration of molecules over the bulk, which increases much more slowly for adsorbate weakly attracted to the adsorbent, that is, lower value of *K*. The adsorption of molecules onto microplastics significantly increases their local concentration. Therefore, microplastics with preadsorbed matter such as heavy metals can be potentially toxic due to the large amount of surface-adsorbed ions. Such aspects of adsorption of molecules/ions onto microplastics should be considered while assessing their potential environmental and health impacts.

The surface chemistry of microplastics governs their interaction with external interfaces and could drive the adsorption of contaminants on its large surface area. This is a topic of concern due to the increasing presence of persistent organic pollutants (POPs) such as perfluoroalkyl substances (PFAS) and polycyclic aromatic hydrocarbons (PAHs) in aquatic environments.^34,35^ Reports have shown that such compounds are found on microparticles at concentrations several orders of magnitude higher than those in surrounding environments.^36,37^ The specific mechanisms of adsorption are still to be investigated with respect to the various environmental parameters involved, such as temperature, pH, and salinity. There is growing evidence indicating that microplastics are ingested by marine organisms, and the implications regarding exposure to organic pollutants are being debated.^38^ There is still a wide knowledge gap, as indicated by the United Nations Environment Programme, regarding the toxicity of microplastics, but initial studies have looked at the possible interactions between particles and cells/tissues.^39^

## Interparticle Interactions

**Figure 4:**
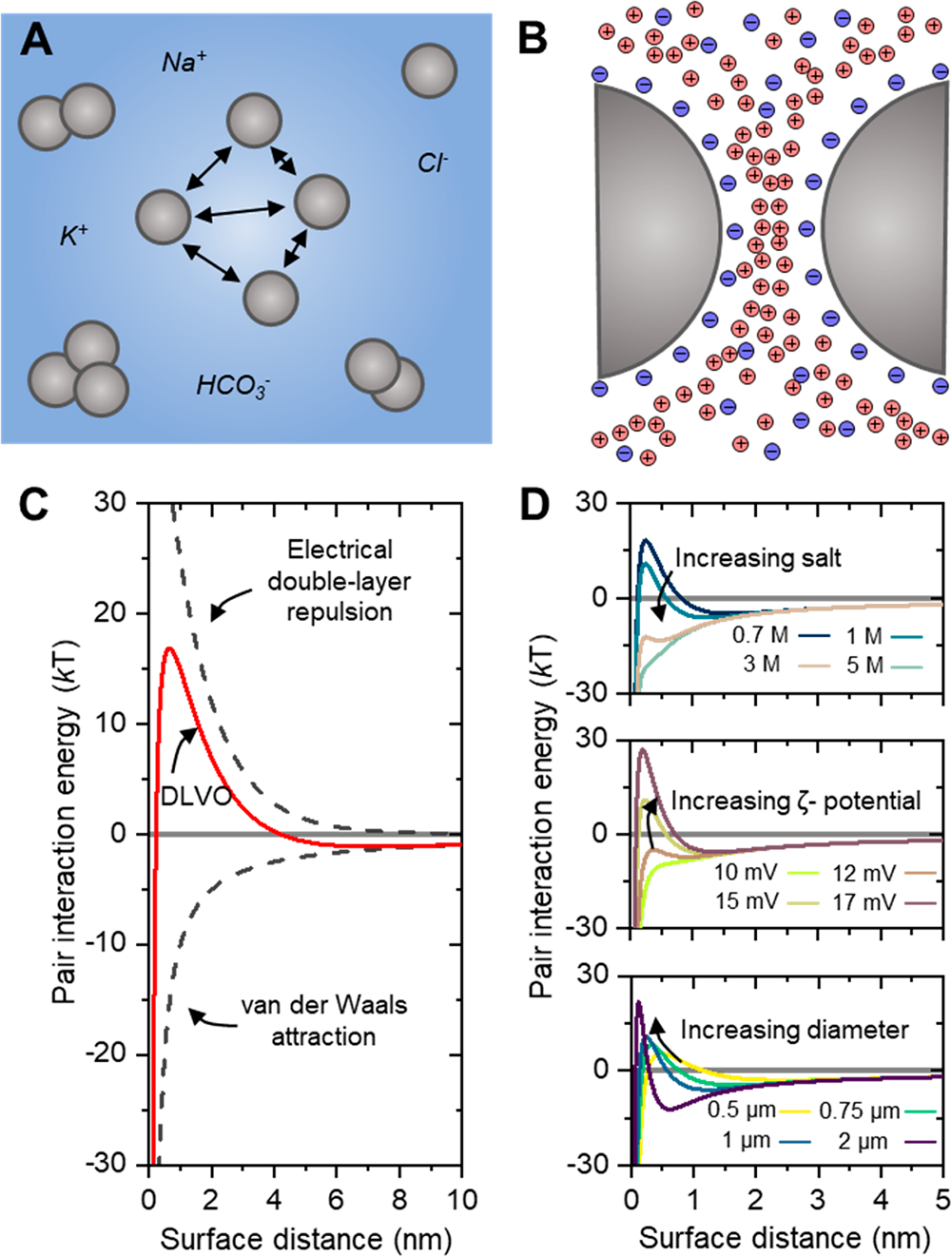
(A) Schematic of colloidal interactions between microplastics across water medium. (B) Electrostatic repulsion resulting from the overlap of counterion clouds. (C) Sample DLVO plots of interaction energy between two 1 μm sized particles. Electrostatic repulsion (positive half) is summed with van der Waals attraction (negative half) to yield a DLVO curve. (D) Increasing salt decreases repulsion. Increasing ζ-potential and/or size increases repulsion.

Colloidal particles are characterized by a number of potential interactions occurring in solution (Figure 4A). The net effect of such interactions can be attraction or repulsion between particles depending on several factors, from particle shape and size to chemical composition of the medium. Therefore, the nature of interactions among colloidal microplastics is key in determining their behavior in the environment. Attraction among particles can lead to their aggregation, while repulsive forces can keep colloids stable with respect to their long-term distribution in the suspension. The most relevant interactions are the ever-present van der Waals attraction and electrical double layer repulsion, which are traditionally described by the theory of Derjaguin–Landau–Verwey–Overbeek (DLVO). The van der Waals interactions arise from the combined effects of weak electric intermolecular forces. These are attractions between two permanent dipoles, a permanent dipole and an induced dipole, or two induced dipoles. Concurrently, particles experience osmotic repulsion due to the overlap of electric double layers with same charge. Briefly, surfaces in water become charged usually due to ionization or dissociation of surface groups. Such surface charge is balanced by an equivalent oppositely charged region of counterions in a diffuse “cloud”. Together, the layer of ions on the particle surface and the counterion cloud form the electric double layer^40^ (Figure 4B). DLVO theory describes the stability and aggregation behavior of colloids in solution based on the resulting interaction obtained by summing van der Waals attraction and double layer repulsion (Figure 4C). Generally, van der Waals forces promote aggregation between colloidal particles that are very close to each other; that is, the attractive interaction is short-ranged in nature. The interaction between two particles of radius *R*_1_ and *R*_2_ originating from van der Waals forces is expressed as , where *D* is the interparticle distance and *A* is the Hamaker constant. This is a coefficient that accounts for the various van der Waals forces at play and is in the order of 10^–20^ J.^41^

Double layer repulsion can prevent aggregation and promote the stability of particles when acting at longer ranges at a safe distance from the surface. The electrostatic interaction energy between two particles in solution is given by , where *Z* is a coefficient analogous to *A* and incorporates the properties of the surface and κ is the inverse of the Debye length. This parameter indicates the “thickness” of the double layer and is a function solely of the solution properties.^40^ This shows that the range of effectiveness of such repulsion is highly dependent on the physicochemical composition of the medium. Therefore, stability or aggregation of microplastics in aquatic environments will strongly depend on the salinity, pH, and temperature of the water.^42^ The net DLVO interaction between a pair of microplastics/particle is calculated as *U*_DLVO_ = *U*_EDL_ + *U*_vdW_.^43^ As shown in Figure 4D, higher salt concentrations favor aggregation because an increase in ion concentration in the medium acts to neutralize Coulombic repulsion between the surface charge and the counterion cloud. This compresses the double layer, thus “screening” the repulsion. Similarly, changes in pH affect the net distribution of charge in the double layer, modifying the so-called zeta-potential (ζ), which is an indirect measure of the ability of colloidal suspensions to remain stable and is quantitatively related to coefficient *Z* used in estimation of *U*_EDL_. Changes in the equilibrium concentration of H^+^ and OH^–^ groups modifies the surface chemistry of particles by interfering with their ionization and dissociation, often reducing the ζ-potential and favoring aggregation.

Traditional DLVO is not always successful in predicting the net effect of interactions, but it provides a baseline theoretical approach that can be extended for specific particle systems and/or conditions.^44^ For example, colloids with apparent electrostatic repulsion sometimes aggregate due to discrete nanoscale surface heterogeneity.^45,46^ This recently developed theory indicates that mean-field DLVO interactions can overgeneralize the surface characteristics of a particle by ignoring patches where seemingly repulsive particles may attract.^47^

Depending on the size and surface properties of the particles as well as the physicochemical properties of the water, microplastics may form homoaggregates as they assemble with each other or heteroaggregates as they combine with other debris or sediment.^48^ The latter case has been found to be relevant in the formation of composites in the presence of other colloids, both inorganic and biological.^49^ Aggregation will lead to sinking when the density of the aggregate is higher than that of water. Conversely, particles lighter than water will have a higher buoyancy when aggregated. DLVO is helpful in describing the tendency of some microplastics to aggregate into clumps and subsequently settle^50^ or showcase stability in river water.^51^

## Colloidal Transport

Several modes of transport are ultimately responsible for the occurrence and deposition of microplastics throughout the environment. Transport can be short-ranged with respect to the source of the microplastics (e.g., sinking or entrainment of particles near waste sites), or it can be long-ranged when it involves fluvial or oceanic currents moving particles at a far distance from their original sites. In addition, the transport of colloidal and granular particles can be predominantly vertical, horizontal, or characterized by a complex interchange of both modes. The main factors driving transport of colloidal and granular materials in aquatic environments are water flows and currents, combined with gravity.^52^ It is important to note that microplastics are often transported across the total environment, including the atmosphere. Presence of microplastics in remote locations is attributed to this latter mode of transport.^4^

In the hydrosphere, the movement of particles depends on their characteristics and the dynamics of the water. The primary distinction in assessing microplastics transports comes from the interplay of gravitational and flow forces. On one hand, particles that are stable against gravity will remain suspended in water and are primarily transported horizontally with its flow. On the other hand, particles (or clusters) that lose buoyancy will tend to sink to the bottom of their reservoir. In this case, horizontal motion is expected to follow a type of bed-load transport.^53^

**Figure 5:**
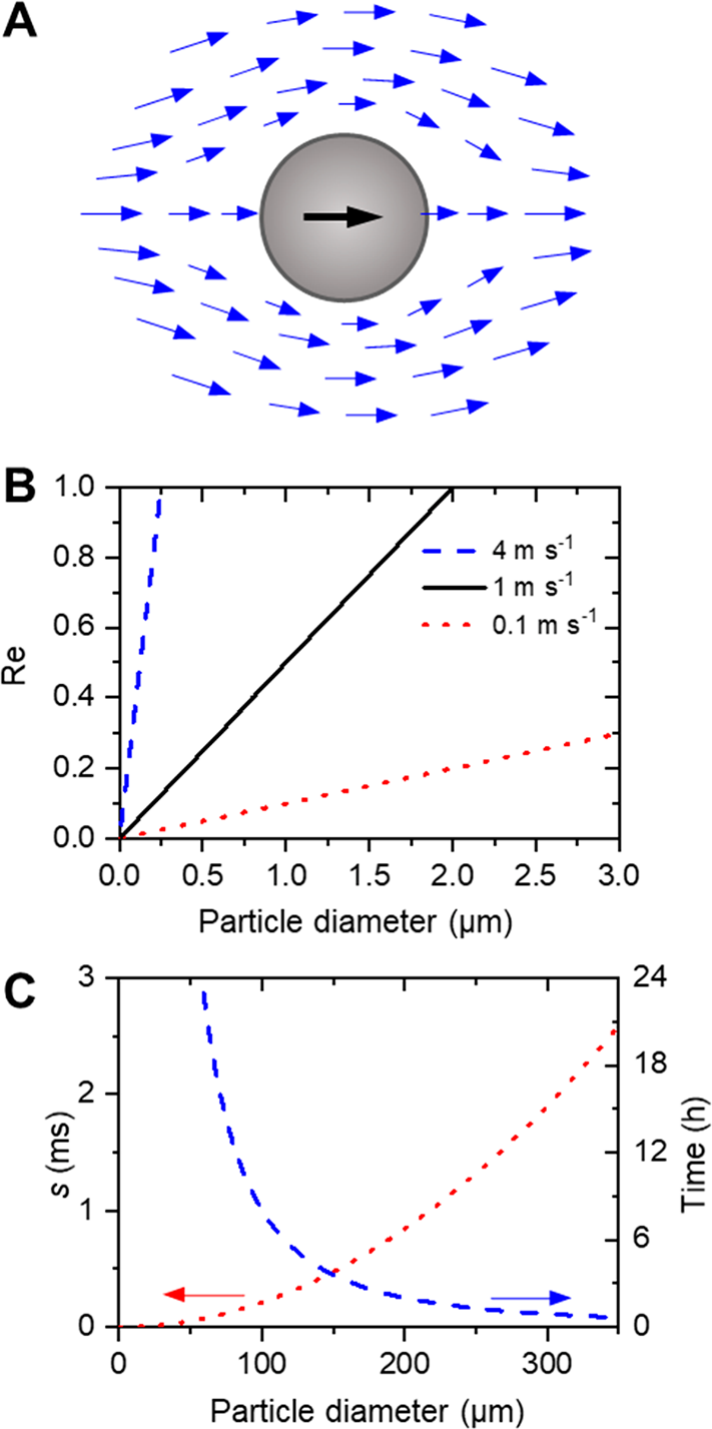
(A) Schematic of Stokes flow around a particle. (B) Change in Reynolds number with microplastics size for different velocities corresponding to average ocean current (*v* = 4 m s^–1^), fast river (*v* = 1 m s^–1^), and slow river (*v* = 0.1 m s^–1^). (C) Sedimentation coefficient as a function of microplastics size (left *y*-axis) and associated time for settling down a 1 m depth (right *y*-axis) for a particle with a density of 1.38 g cm^–3^.

The ratio of inertial and viscous forces in fluid flow determines the framework of analysis for transport. This is determined by the dimensionless value of the Reynolds number, , which relates inertial and viscous forces based on the density of the fluid ρ_f_, its velocity *v*, its viscosity η, and the characteristic size of the immersed object *l*. The Reynolds number is directly proportional to *l*, indicating that microplastics are less affected by inertial forces compared to macroplastics. Effectively, microscopic objects experience water similarly to how large ones experience honey; that is, they move in slow viscous flows. Such fluid motions are described using Stokesian dynamics which may help in predicting the pathway and distance covered by microplastic debris in slow rivers and ocean currents in which Re ≪ 1 (Figure 5A). In a river flowing at 0.1 m s^–1^, a 3 cm bottle cap would experience turbulent flow with a Re of ∼3000. In the same river, the Re of a suspended microparticle of 1 μm in diameter would be 0.1, falling in the Stokesian regime (Figure 5B).

In the absence of horizontal flows, the settling and sedimentation of microplastics is described using Stokes’ law; that is, the drag force on a particle of radius *R* is *F* = 6π*R*η*v*. This is used in expressing the sedimentation coefficient *s* of a spherical particle as a function of the density gradient, , where ρ is the density of the particle and *s* is a useful parameter in estimating the time required for particles to travel down the water column.^54^ This is important in nonflowing aquatic environments such as lakes, swamps, and near dams. The time required for an object to sink down a depth *z* is inversely proportional to the sedimentation coefficient. This time may be estimated as , where *g* is the gravitational acceleration. Microplastics are more stable in water compared to macroplastics; that is, their values of *s* are small, yet even with *s* = 0.2 ms, a 100 μm particle will sink by 1 m every 8 h (Figure 5C). This indicates that in oceans, particles that are not buoyant will eventually settle such that the sea floor is expected to house a large volume of microplastic debris.^55^

Bed-load transport is traditionally studied for granular sediment and is related to the motion of particles as they roll or slide along a reservoir bed.^56^ This transport mode may play a unique role for microplastics. For example, rivers are found to act as both a sink and a source of microplastic debris due to the dynamic behavior of the “active layer” of the sediment bed.^57^ This is defined as the stratum involved in mass exchange with the water column, which changes dramatically during flooding events. It was recently shown that the response of the sediment bed to changing hydraulics during floods transforms rivers from sinks to sources of microplastics.^57^ Our fundamental understanding of the physics of colloidal transport in porous media can provide valuable insight into the study of microplastics in such dynamic conditions.^47,58,59^ Specifically, it can help in predicting the extents of entrainment and release of plastic particles that occur as wet and dry periods cycle over time. This is highly relevant not only in riverbanks, but other bodies of water, as well. Tidal waves affect the distribution of microplastics in coastal seas as the water transports particles to beaches or resuspends trapped particles.^60^

The relative importance of horizontal and vertical flows is nontrivial, particularly in estuarine waters, making predictions regarding the transport of microplastics much more difficult. It should also be noted that most plastics have densities between 0.8 and 1.5 g cm^–3^, which are closer to water density compared to sediment material. This indicates that applying existing models of sediment transport to microplastics should be done with great care.

## Overall Picture

Wetting, adsorption, particle interactions, and transport are colloidal phenomena that span countless fields of research, including environmental science. Microplastics, due to their size and surface properties, are (mostly) water-based colloids whose behaviors can be understood under the lens view of colloid and interface science. Each of the aspects addressed here displays its own scientific challenges. For example, wetting and adsorption are often taken as predefined behaviors when in fact they depend on dynamic properties, namely, the changing surface chemistry of particles. The interactions that particles experience across media are only approximately described by DLVO theory: some major deviations are expected because of discrete nanoscale surface heterogeneity and hydrophobic/solvation effects. Microplastic transport is a complex phenomenon that may not be deterministically predictable and analytical efforts may be limited to specific circumstances.

At the same time, many physicochemical properties and dynamics of microplastics are coupled: it is inevitable that surface chemistry affects interparticle interactions, which affect transport and sedimentation. The value of adopting the framework of colloid science is in the holistic view that it offers. The perspective of microplastics as a colloid provides a scientifically established template for understanding the environmental challenge both through a priori predictions and a posteriori analysis. This type of overall view based on sound basic science forms a solid foundation on which to build mitigation and remediation plans and policies.

Many potential topics of research are outlined above, from studying the adsorption of pollutants on microplastics to their flow-through porous media. Another area with important questions is the interaction of microplastics with biological organisms, including biofilm formation. The principles of colloid and interfacial science may act as a juncture connecting the environmental science revolving around microplastics with the physical sciences of small things.
